# Modification of pectoral fins occurs during the larva-to-juvenile transition in the mudskipper (*Periophthalmus modestus*)

**DOI:** 10.1186/s40851-018-0105-z

**Published:** 2018-08-11

**Authors:** Eri Okamoto, Hieu Van Mai, Atsushi Ishimatsu, Mikiko Tanaka

**Affiliations:** 1 School of Life Science and Technology, Tokyo Institute of Technology, B-17, 4259 Nagatsuta-cho, Midori-ku, Yokohama, Japan; 2 Graduate School of Fisheries and Environmental Sciences, Nagasaki University, 1-14 Bunkyomachi, Nagasaki, 851-8521 Japan; 3 Institute for East China Sea Research, Organization of Marine Science and Technology, Nagasaki University, 1551-7 Tairamachi, Nagasaki, 851-2213 Japan

**Keywords:** Mudskipper, Pectoral fin, Development

## Abstract

**Background:**

Mudskippers are amphibious fishes that use their pectoral fins to move on land. Their pectoral fins are specifically modified for terrestrial locomotion. Studies of the anatomy and kinematics of adult mudskippers suggest that modifications of the pectoral fins, such as their protrusion and elongation of the proximal radials, may provide greater control and flexibility in pectoral fin–based locomotion. However, it is unknown when and how the unique features of these pectoral fins form during the development of mudskippers, which begin life as a planktonic organism.

**Results:**

Here we examined the developmental process of the pectoral fins of the mudskipper *Periophthalmus modestus* to address these questions. We also observed other developmental characteristics to provide clarified descriptions, including indicative morphological changes that occur during metamorphosis.

**Conclusion:**

Our results show that the localized cell division of the proximal part of the endoskeletal disc—the primordium of the proximal radials—and subsequent cell division along the proximal-distal axis, which is restricted to the distal part of the disc during the larva-to-juvenile transition (metamorphosis), lead to the elongation of the proximal radials.

**Electronic supplementary material:**

The online version of this article (10.1186/s40851-018-0105-z) contains supplementary material, which is available to authorized users.

## Background

Mudskippers (family Gobiidae: subfamily Oxudercinae) are amphibious teleosts. Of 10 oxudercine genera, the species in *Periophthalmus* and *Periophthalmodon* are the most terrestrial, spending much of their time on land [1]. Both their distinctive morphology and physiology are the result of evolutionary adaptations for life on mudflats. In particular, the morphology of their pectoral fins has been drastically modified to allow them to “walk” in a semi-terrestrial environment [1].

Endoskeletal elements of the pectoral fins of *Periophthalmus* exhibit unique morphological characteristics [2, 3]; the proximal radials are more elongated and protrude farther from the body wall than in other gobies [2]. This unique skeletal pattern allows the creation of two movable hinge joints at the boundaries between the cleithrum and the proximal radials and between the proximal radials and the fin rays, which presumably facilitates to aid the terrestrial locomotion of these amphibious fishes [2, 4]. Elongation of the proximal radials also provides a larger area onto which pectoral muscles can attach [2]. In addition to the elongated proximal radials, mudskippers have enlarged cleithrum plates to which pectoral muscles attach, thereby helping to create force and motility to support their body weight [2, 5]. These anatomical features and physiological functions of mudskipper pectoral fins have been studied intensively [1–5]; however, it remains uncertain how and when such morphological modifications develop during the embryogenesis of these fishes.

**Fig. 1:**
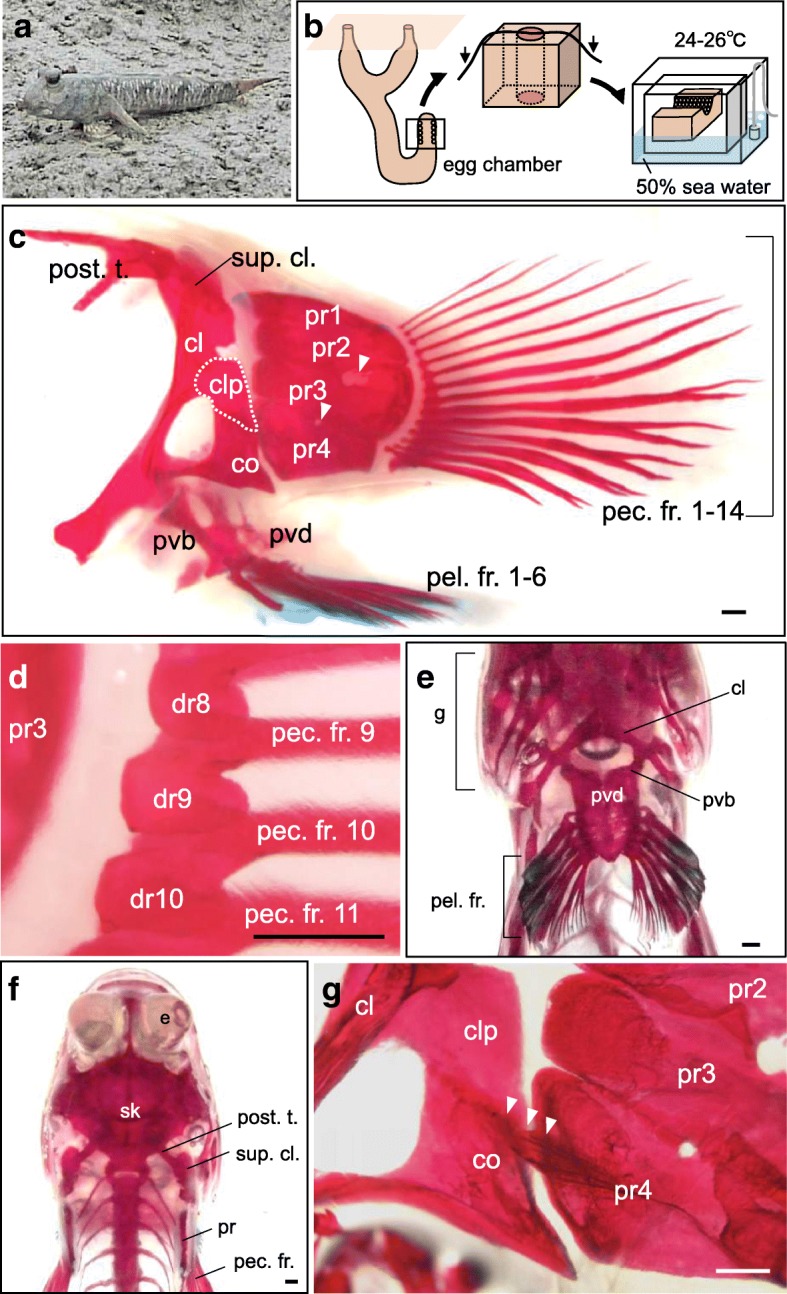
Collection of samples and the pectoral fin of an adult mudskipper *Ps. modestus*. **a** The adult mudskipper (*Periophthalmus modestus*). **b** The egg chamber and surrounding mud was excavated, transferred to a humid container, and brought back to the laboratory. The open container was set adrift in an aerated aquarium kept within an incubator. **c**–**g** Skeletal patterns of an adult mudskipper (*n* = 1). **c** Lateral view of the pectoral fin of an adult mudskipper. Arrowheads indicate inter-radial fenestrae. **d** Ventral view of the medial part of the pectoral fin. **e**, **f** Ventral (**e**) and dorsal (**f**) views of the thoracic region of an adult mudskipper. **g** Ventral view of the proximal part of the pectoral fin. The fourth proximal radial partially articulates with the coracoid (arrowheads). cl, cleithrum; clp, cleithrum plate; co, coracoid; dr1–14, distal radials 1 to 14; e, eye; g, gill; pec. fr. 1–14, pectoral fin rays 1 to 14; pel. fr. 1–6, pelvic fin rays 1 to 6; post. t., posttemporal; pvb, pelvic bone; pr1–4, proximal radials 1 to 4; pvd, pelvic dome; sk, skull; sup. cl., supracleithrum. Scale bars, 1 mm

The mudskipper *Ps. modestus* (Fig. 1a) inhabits mudflats in Japan and lays its eggs in mud burrows. The male transfers mouthfuls of air into the egg chamber until embryo development is complete [6]. Egg hatching is induced when the male removes air from the chamber and releases it outside of the burrow [6]. Hatched larvae spend 30 to 50 days in plankton before they start to live in semi-aquatic environments.

Here we examined the development of the pectoral fins of the mudskipper *Ps. modestus* and explored when and how their unique pectoral skeletal patterns, such as the protrusion of the girdle and the elongation of the proximal radials, are formed. Our results show that the localized cell division of the proximal part of the endoskeletal disc, which is the primordium of the proximal radials, and subsequent cell divisions of chondrogenic cells along the proximal-distal axis only in its distal region during the larva-to-juvenile transition lead to the elongation of the proximal radials.

## Methods

### Embryos, larvae (total length [TL], ~ 5.6 mm), and adults

Mudskipper (*Periophthalmus modestus*) eggs and adults were collected from mudflats at the mouth of the Fukushoe River, Saga Prefecture, Japan on 20 June, 2016. Adults were captured with a net. For egg collection, the mud over an egg chamber and the main shaft was excavated down to the level of the roof of the egg chamber (Fig. 1b, left). When the chamber was exposed and the fertilization of the eggs was verified, the egg chamber and its surrounding mud were completely excavated, cut in half by a thread (Fig. 1b middle), and transferred to a container with a moist paper towel soaked in 50% seawater for transport back to the laboratory (Fig. 1b right). We then set the open container (with eggs and mud inside) adrift in an aerated aquarium (50% seawater) in an incubator at 24–26°C (Fig. 1b right). When the embryos reached the stage just before hatching [7], the eggs were immersed in 50% seawater in the aquarium kept in the incubator at 24–26°C to induce hatching. Hatched larvae were then kept in rearing water (50% seawater with marine chlorella) and fed with rotifers twice a day.

To fix the embryos, bundles of adhesive filaments on the eggshell [7] were removed, and surface mud was removed with several washes in distilled water. Eggs and hatched larvae were fixed in 4% paraformaldehyde (PFA) overnight at 4°C and were then washed in phosphate-buffered saline containing 0.1% Tween 20 (PBT). Fixed eggs were de-chorionated. Adults were fixed in 10% formalin and stored at 4°C. Fixed specimens (embryos and larvae) were dehydrated in a graded methanol/PBT series and stored in 100% methanol at − 20°C. Specimens were rehydrated in a graded methanol/PBT series and observed under a stereomicroscope (Leica MZ16F) and an upright microscope (Zeiss Axioskon).

### Larvae (TL, 6.2 mm~) and juveniles

Specimens were collected on 17 August, 2015, and reared as described above. Hatched larvae were fed with rotifers until they were 20 days old and were subsequently fed with rotifers and Artemia. Specimens were fixed in 10% formalin and stored at room temperature. They were washed in PBT before observation. Some fixed specimens were stained with alcian blue and alizarin red as described [8]. Both stained and non-stained specimens were observed as described above.

### Histology

Specimens were prepared as described [9] with modifications. Briefly, methanol-preserved specimens were re-hydrated in a graded methanol/PBT series. These specimens and formalin-preserved specimens were then washed in PBT and dehydrated in a graded ethanol/PBT series. The 100% ethanol was then replaced with 100% acetone, and the specimens were embedded in Technovit 8100 resin (Heraues-Kulzer). Sections were cut at a thickness of 10 μm, stained with hematoxylin, washed in tap water, stained with eosin, washed in tap water, treated with xylene and covered in Mount Quick (Daido Sangyo).

## Results

### Adult fin skeletons

First, we examined the pectoral skeletal pattern of adult fish. The pectoral girdle connects the pectoral fins to the axial skeleton. It consists of the post-temporal (post. t.), supracleithrum (sup. cl.), cleithrum (cl), and coracoid (co) (Fig. 1c). The girdle supports four proximal radials (pr1–pr4) (Fig. 1c). Fourteen distal radials are located distal to the proximal radials (Fig. 1d), and 14 exoskeletal fin rays are aligned at the distal part of the pectoral fin (pec. fr. 1–14) (Fig. 1c).

The cleithrum, the main element of the pectoral girdle, is a dorsoventrally elongated bone located at the base of the pectoral fin (Fig. 1c). The cleithrum becomes wider in the middle, at which point it is referred to as the cleithrum plate (clp in Fig. 1c), as in *Ps. barbarus*, *Pn. schlosseri*, and *Ps. argentilineatus* [2, 4, 5]. The ventral edge of each of the bilateral cleithra is located behind the gill, and their surfaces are opposed at the midline (Fig. 1e). The ventral part of the cleithrum adjacent to the coracoid connects to the pelvic bone (pvb in Fig. 1c, e), and pelvic domes (pvd) at the edge of each pelvic bone fuse at the midline (Fig. 1e). The dorsal part of the cleithrum connects to the occipital region via the post-temporal and the supracleithrum (Fig. 1f). The coracoid is situated ventrally to the cleithrum plate (Fig. 1c) and partially articulates with the fourth proximal radial (arrowheads in Fig. 1g), as in *Ps. barbarus* [2].

Each proximal radial partially overlaps and looks like a large single plate (pr1–pr4 in Fig. 1c), but small spaces are recognized between the second and the third, as well as between the third and the fourth, proximal radials (arrowheads in Fig. 1c). The first proximal radial (pr1) is a long, thin bone. The fourth proximal radial (pr4) is the shortest element among the four proximal radials (Fig. 1c), as reported in *Ps. barbarus* [2]. The first to eighth fin rays are segmented soft rays, whereas the ninth to fourteenth fin rays are bifurcated bony rays (Fig. 1c).

### Development of the pectoral fins and other developmental characteristics

To explore when and how pectoral fins of the mudskipper *Ps. modestus* become elongated and protrude from the body wall, we observed the developmental process of the pectoral fins. Although *Ps. modestus* development has been described for embryos and larvae obtained by artificial fertilization [7], more detailed descriptions are required for accurate understanding of the developmental process. Therefore, we also observed the developmental characteristics of embryos upon arrival at the laboratory, which were at the otic vesicle stage (Additional file 1: Figure S1A-C), a stage equivalent to the 39- to 46-h post fertilization (hpf) stage described in Figure 1L in Kobayashi et al. (1972). The developmental stages and elapsed time of each stage are summarized in Additional file 2: Table S1.

#### Embryonic stages (Fig. 2; Fig. 3)

##### Otic vesicle stage (Additional file 1: Figure S1A-C; “39–46 hpf-stage, Figure 1L in Kobayashi et al., 1972”)

During this stage, the tail of the embryo separates from the yolk (Additional file 1: Figure S1A). The otic placodes hollow and form the otic vesicles (ov), and the optic primordia with the lens placodes (lp) are also observed (Additional file 1: Figure S1B). There are mesenchymal cells (mc) at the distal end of the tail (Additional file 1: Figure S1C). The heart (h) is visible (Additional file 1: Figure S1B), but not beating.

##### Brain vesicle stage (Additional file 1: Figure S1D-F)

The heart begins to beat at this stage (Additional file 1: Figure S1D). The tail elongates (Additional file 1: Figure S1D). The brain vesicles, such as the telencephalon (t) and diencephalon (d) of the forebrain, the midbrain (m), and the rhombomeres (r) of the hindbrain, are visible (Additional file 1: Figure S1E). Xanthophores appear in the head and the caudal part of the trunk (open arrowheads in Additional file 1: Figure S1E, F). Two otoliths (ot) are recognized within each otic vesicle (Additional file 1: Figure S1E’). Melanophores appear laterally on the trunk and tail (arrowheads in Additional file 1: Figure S1F). The anus is formed at the posterior end of the gut (black arrow in Additional file 1: Figure S1F).

##### Heart chamber stage (Additional file 1: Figure S1G-I)

The heart tube is divided into the atrium (at) and the ventricle (vt) (Additional file 1: Figure S1H). Mesenchymal cells at the distal end of the tail have disappeared by this stage (Additional file 1: Figure S1I). Xanthophores spread in the head and along the ventral and dorsal edges of the trunk (Additional file 1: Figure S1G’).

##### Circulation stage (Additional file 1: Figure S1J-K)

Blood cells are first detected during this stage (black arrowheads in Additional file 1: Figure S1K). The blood from the heart circulates throughout the body and is collected via the common cardinal vein (ccv in Additional file 1: Figure S1K).

##### Eye pigmentation stage (Fig. 3a; Additional file 1: Figure S1L-M)

At this stage, melanophores are noticeable only at the posterior edge of the optic vesicles (arrowheads in Additional file 1: Figure S1M). Some xanthophores are also seen in the optic vesicles (open arrowheads in Additional file 1: Figure S1M). Cross-sections of an embryo at the presumptive fin bud level show the thickening of the somatopleure, which consists of two or three layers of mesenchymal cells (Fig. 3a). The overlying ectoderm forms the single-cell-layered apical ectodermal thickening (Fig. 3a).

**Fig. 2:**
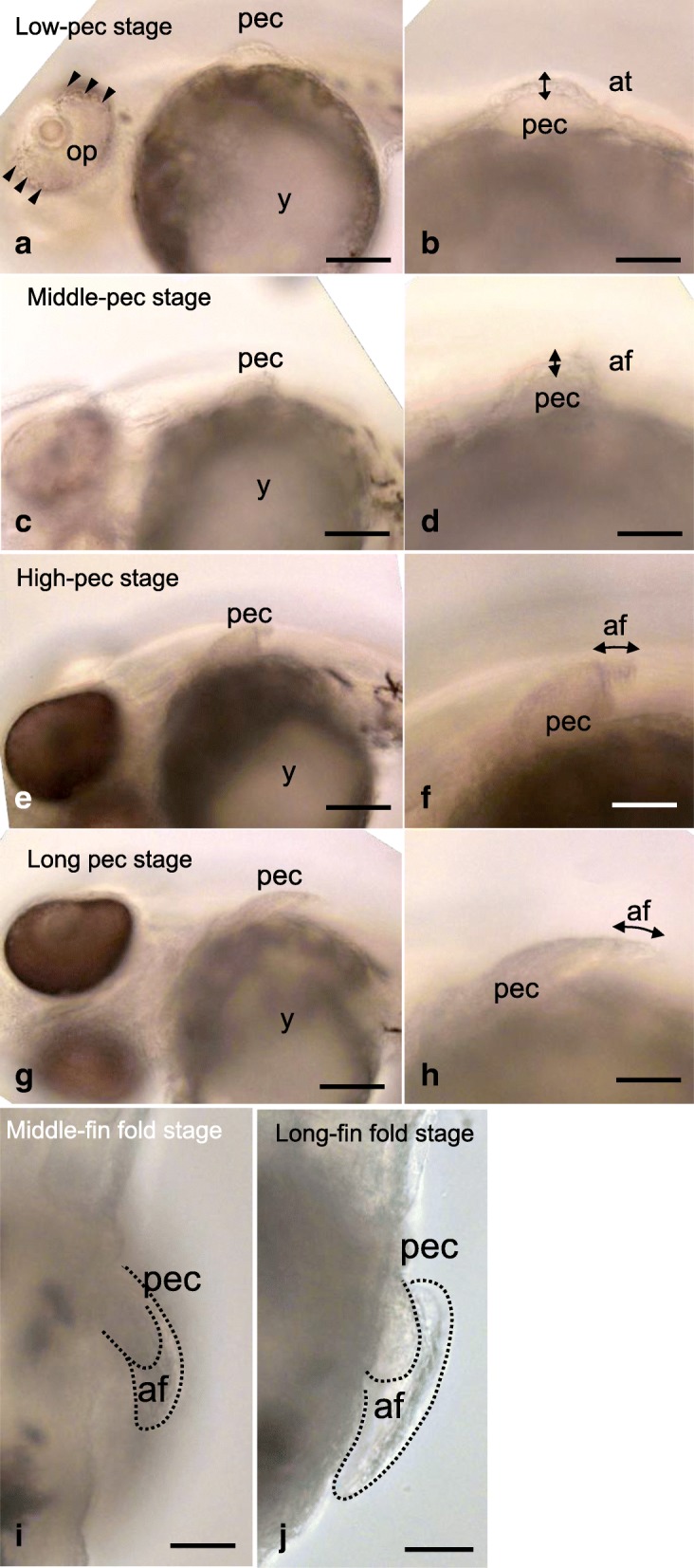
Development of the pectoral fin bud of mudskipper embryos prior to the hatching stage. Ventrolateral oblique views of mudskipper embryos (**a**, **c**, **e**, **g**) and lateral view of pectoral fin buds (**b**, **d**, **f**, **h**) at the low-pec stage (**a**, **b**; *n* = 1), middle-pec stage (**c**, **d**; *n* = 1), high-pec stage (**e**, **f**; *n* = 1), and long-pec stage (**g**, **h**; *n* = 1). Arrowheads in (**a**) indicate melanophores. Dorsal view of pectoral fin buds at the middle fin-fold stage (**i**; *n* = 1) and long fin-fold stage (**j**; *n* = 1). at, apical thickening; af, apical fin fold; op, optic vesicle; pec, pectoral fin. Scale bars, 100 µm in (**a**, **c**, **e**, **g**); 50 µm in (**b**, **d**, **f**, **h**, **i**, **j**)

##### Low-pec stage (Fig. 2a, b)

At this stage, pectoral fins are budding on the surface of the yolk (Fig. 2a), and the apical ectodermal thickening is present at the tip of the pectoral fin bud (Fig. 2b). Melanophores are now seen at both the anterior and posterior edges of the optic vesicles (arrowheads in Fig. 2a).

**Fig. 3:**
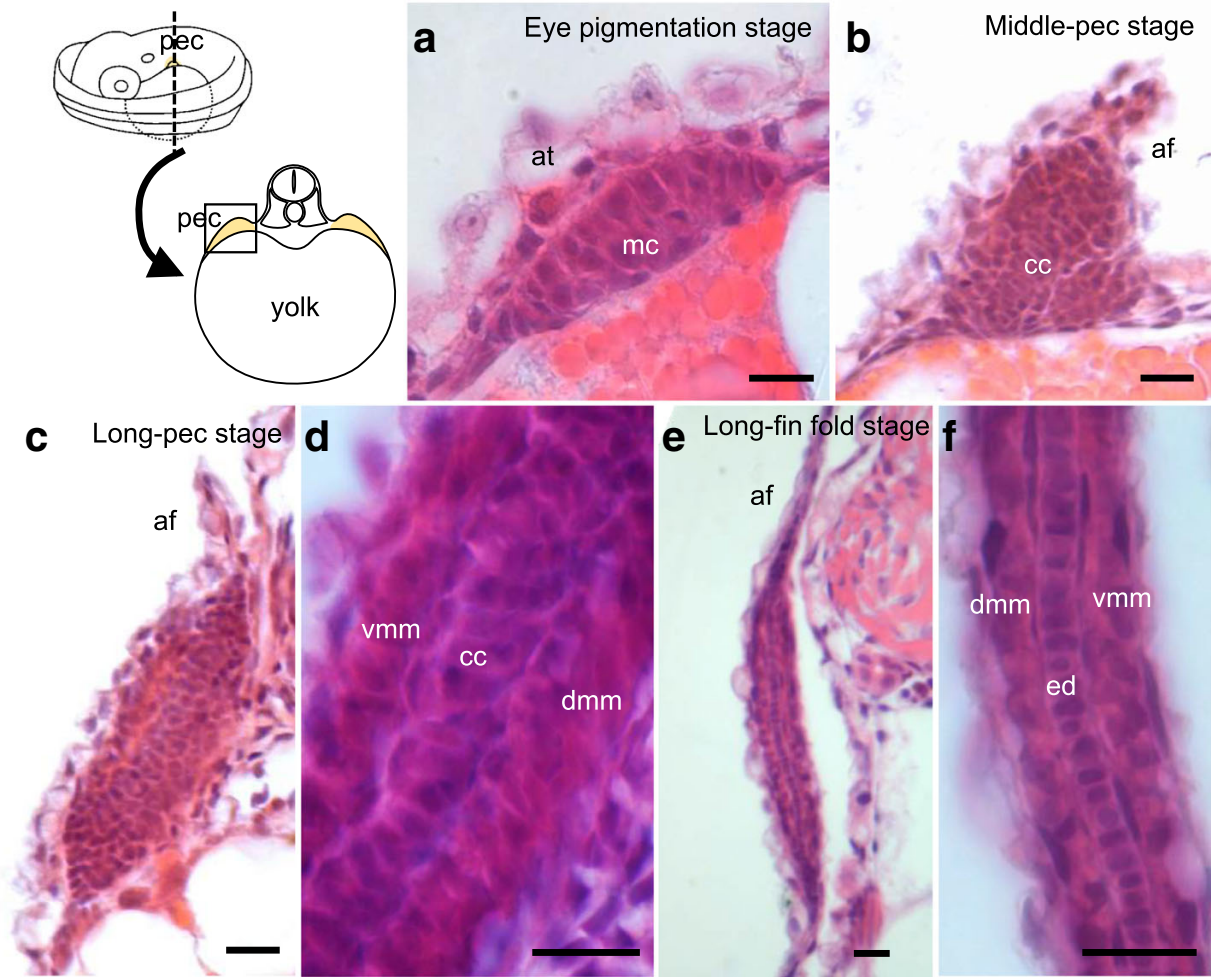
Hematoxylin-eosin staining of pectoral fin buds and early larval fins. Cross-sections of pectoral fin buds and early larval fins stained with hematoxylin and eosin (**a**, **b**, **c**, **e**) and magnified images (**d**, **f**) at the eye pigmentation stage (**a**; *n* = 1), middle-pec stage (**b**; *n* = 1), long-pec stage (**c**, **d**; *n* = 1), and long fin-fold stage (**e**, **f**; *n* = 1). at, apical thickening; af, apical fin fold; cc, chondrogenic condensation; dmm, dorsal muscle mass; ed., endoskeletal disc; vmm, ventral muscle mass; pec, pectoral fin. Scale bars, 10 μm

##### Middle-pec stage (Fig. 2c-d; Fig. 3b; Additional file 1: Figure S1N-O)

The height of the pectoral fin bud is approximately half the length of its width (Fig. 2c, d). Pigmentation is noticeable throughout the eye (Additional file 1: Figure S1N). The swimbladder (sb) begins to be inflated (Additional file 1: Figure S1O). A cross-section of the pectoral fin bud shows the transformation of the apical ectodermal thickening into the two cells layered apical fin fold (Fig. 3b). A chondrogenic condensation becomes visible in the center of the fin bud (Fig. 3b).

##### High-pec stage (Fig. 2e-f; Additional file 1: Figure S1P, Q)

At this stage, the pectoral fin bud curves posteriorly and tapers (Fig. 2e, f). The apical ectodermal thickening transforms into the apical fin fold (af in Fig. 2f). Pigmentation of the eye is very dense (Fig. 2e). Rotation of the heart tube is noticeable (Additional file 1: Figure S1Q).

##### Long-pec stage (Fig. 2g, h; Fig. 3c, d)

During this stage, the pectoral fin bud elongates parallel to the body axis (Fig. 2g, h), suggesting its rotation is completed. In the cross-section of the pectoral fin bud (Fig. 3c), the apical fin fold elongates, and the chondrogenic cells (cc) align along the proximal-distal axis and divide the pectoral muscles into dorsal and ventral muscle masses (dmm and vmm, respectively) within the pectoral fin buds (Fig. 3d).

##### Middle fin-fold stage (Fig. 2i)

The pectoral fin bud and the apical fin fold further elongate during this stage. The length of the apical fin fold now makes up two-fifths of the total length of the bud (Fig. 2i).

##### Long fin-fold stage (Fig. 2j; Fig. 3e, f; Additional file 1: Figure S1R-T)

The length of the apical fin fold now makes up three-fifths of the total length of the bud (Fig. 2j). The mouth opens (Additional file 1: Figure S1S), and the otic vesicles develop their characteristic inverted-heart shape (Additional file 1: Figure S1S, S′). Both the dorsal median fin fold and the ventral median fin fold begin to narrow in the caudal region (arrows in Additional file 1: Figure S1T). In a cross-section of the pectoral fin bud, cuboidal chondrogenic cells are seen to align to form a single-cell-layered endoskeletal disc in the center of the pectoral fin bud (Fig. 3e, f).

##### Hatching stage (Additional file 1: Figure S1U-V)

The upper jaw (uj) now protrudes at the same level as the lower jaw (Additional file 1: Figure S1V).

#### Planktonic larval stages (Fig. 4; Fig. 5; Fig. 6)

##### 3.0–4.3 mm TL (Fig. 4a; Fig. 5a-c; Additional file 3: Figure S2A)

**Fig. 4:**
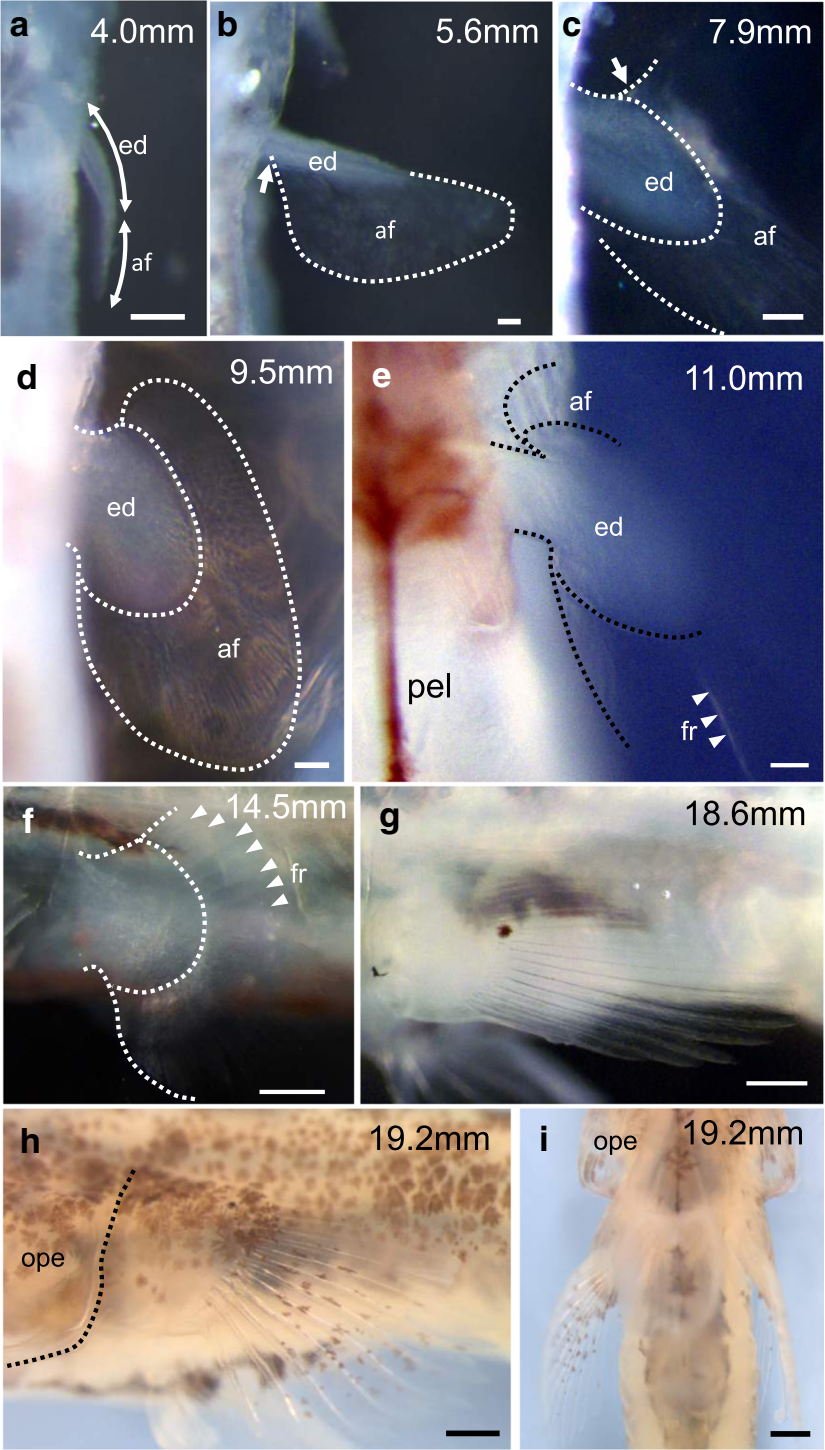
Development of the pectoral fins of mudskipper larvae and juveniles. Dorsal (**a**–**d**), ventral (**e**, **i**), and lateral views (**f**–**h**) views of planktonic larvae at TL 4.0–14.5 mm stages (**a**–**f**; *n* = 1 each stage) and benthic to amphibious juveniles at TL 18.6–19.2 mm stages (**g**–**i**; *n* = 1 each stage). Arrows in (**b**, **c**) indicate the boundary between the endoskeletal disc and the fin fold. Arrowheads in (**e**, **f**) indicate fin rays. af, apical fin fold; ed, endoskeletal disc; fr, fin ray; ope, operculum; pel, pelvic fin. Scale bars, 100 µm in a-e; 500 µm in **f**–**i**

**Fig. 5:**
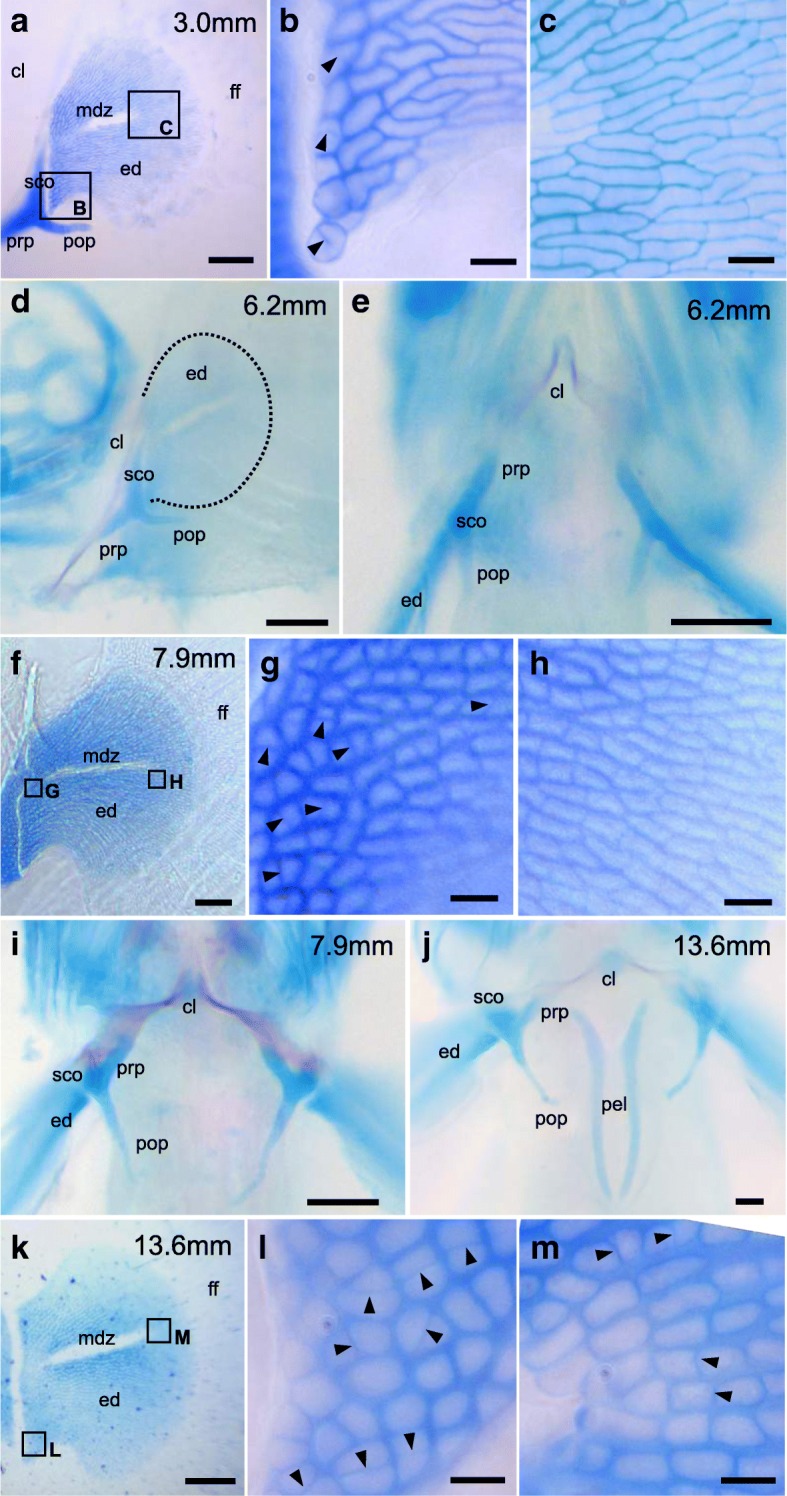
Skeletal patterns of pectoral fins of larvae. Lateral (**a**–**d**, **f**–**h**, **k**–**m**) and ventral views (**e**, **i**, **j**) of pectoral fins of planktonic larvae at 3.0–13.6 mm TL stained with alcian blue and alizarin red (*n* = 1 each stage). **b**, **c**, **g**, **h**, **l**, **m** Magnified views from the indicated images. Arrowheads in (**b**, **c**, **g**, **h**, **l**, **m**) indicate thin matrix septa within dividing cells. cl, cleithrum; ed., endoskeletal disc; ff, fin fold; mdz, zone of matrix decomposition; pop, postcoracoid process; prp, precoracoid process; pel, pelvic bone primordium; sco, scapulocoracoid. Scale bars, 100 μm in (**d**, **e**, **i**, **j**); 50 μm in (**a**, **f**, **k**); 10 μm in (**b**, **c**, **g**, **h**, **l**, **m**)

The size of larvae artificially induced to hatch ranged from 3.0 to 4.3 mm TL (Additional file 3: Figure S2A). The larva at this stage has a round head, a continuous median fin fold, and a yolk sac (Additional file 3: Figure S2A). The endoskeletal disc (ed) and the apical fin fold (af) are located at the proximal and distal regions of the pectoral fin bud, respectively (Fig. 4a). A thin zone of matrix decomposition (mdz) is seen in the center of the endoskeletal disc of the pectoral fin (Fig. 5a). At the proximal part of the endoskeletal disc, cells are more rounded, and small numbers of dividing cells are noticeable (Fig. 5b). At the distal part of the endoskeletal disc, cylindrical cells are aligned along the proximal-distal axis (Fig. 5c). Proximal to the endoskeletal disc, there are chondrified girdle primordia, consisting of the cleithrum (cl), scapulocoracoid (sco), postcoracoid process (pop), and precoracoid process (prp) (Fig. 5a). The skeletal elements of the girdle are formed within the body wall. The cleithrum develops as a thin element (Fig. 5a). The scapulocoracoid is seen in between the cleithrum and the endoskeletal disc (Fig. 5a). The precoracoid process is growing anteriorly, and the postcoracoid process is growing posteriorly (Fig. 5a).

##### 5.6 mm TL (Fig. 4b; Additional file 3: Figure S2B)

The lower jaw protrudes beyond the upper jaw (Additional file 3: Figure S2B). The yolk is completely absorbed (Additional file 3: Figure S2B). The pectoral fin bud further elongates (Fig. 4b). There are no dramatic changes in the shape of the endoskeletal disc (Fig. 4b). The apical fin fold contacts the proximal end of the endoskeletal disc and is located close to the body wall (arrow in Fig. 4b).

##### 6.2 mm TL (Fig. 5d-e; Additional file 3: Figure S2C, D)

The caudal tip of the notochord begins to curve dorsally (arrow in Additional file 3: Figure S2D). There are fin rays at the ventral part of the caudal fin fold (arrowheads in Additional file 3: Figure S2D). Ossification of the cleithrum is observed behind the gill (Fig. 5d, e). No prominent morphological changes are observed in the skeletal elements of the girdle, except for the cleithrum ossification.

##### 7.9 mm TL (Fig. 4c; Fig. 5f; Additional file 3: Figure S2E-F)

Pelvic fin buds (pel) are first recognized as small bulges, and no apical ectodermal thickening is observed (Additional file 3: Figure S2F). Initiation of pelvic fin bud formation is one of the morphological changes occurring during the larva-to-juvenile transition (metamorphosis) in teleost fishes [10]. The median fin forms the second dorsal fin (df2) and the anal fin (af) (Additional file 3: Figure S2E). The caudal region of the round-shaped median fin transforms into the paddle-shaped caudal fin (cf in Additional file 3: Figure S2E). The primordium of the hypural bone, which supports elements of the caudal fin rays, is observed as dorsally curving vertebrae at the caudal edge (arrow in Additional file 3: Figure S2E). The proximal end of the apical fin fold of the pectoral fin begins to move away from the body wall (arrow in Fig. 4c). The proximal part of the endoskeletal disc of the pectoral fin elongates and becomes fan-like in shape (Fig. 4c; Fig. 5f). At the proximal region of the endoskeletal disc, there are cuboidal cells with thin matrix septa (arrowheads in Fig. 5g), indicative of cell divisions [11]. There are cylindrical cells at the distal part of the endoskeletal disc, and no obvious matrix septa are seen in any observed cells (Fig. 5h). The cleithrum is further ossified (Fig. 5i). The angle between the precoracoid process and the postcoracoid process is smaller than that at 6.2 mm TL (compare Fig. 5e, i), which leads to the protrusion of the scapulocoracoid from the body wall (Fig. 5i). The postcoracoid process is further elongated posteriorly (Fig. 5i).

##### 9.5 mm TL (Fig. 4d)

The proximal region of the endoskeletal disc of the pectoral fin is further elongated (Fig. 4d). The fan-shaped pectoral fin is completely separated from the body wall (Fig. 4d).

##### 11.0 mm TL (Fig. 4e; Additional file 3: Figure S2G-H)

The pelvic fin buds become more distinct (Additional file 3: Figure S2H). Melanophores are found on the dorsal and ventral parts of the caudal end of the body trunk (arrowheads in Additional file 3: Figure S2G). There are two fin rays within the apical fin fold of the pectoral fins (arrowheads in Fig. 4e).

##### 13.6 mm TL (Fig. 5j-m)

The scapulocoracoid sticks out from the surface of the body wall and forms the proximal part of the pectoral fins. Chondrogenesis is initiated in the primordium of the pelvic fin skeleton (Fig. 5j). There are cuboidal chondrogenic cells throughout the endoskeletal disc of the pectoral fins (Fig. 5l, m). Some cuboidal cells have thin matrix septa (arrowheads in Fig. 5l, m), suggesting that they are dividing. At the proximal region of the endoskeletal disc, cell divisions are randomly oriented (Fig. 5l), whereas they occur along the proximal-distal axis in the distal region (Fig. 5m).

##### 14.5 mm TL (Fig. 4f; Fig. 6a-c; Additional file 3: Figure S2I-J)

**Fig. 6:**
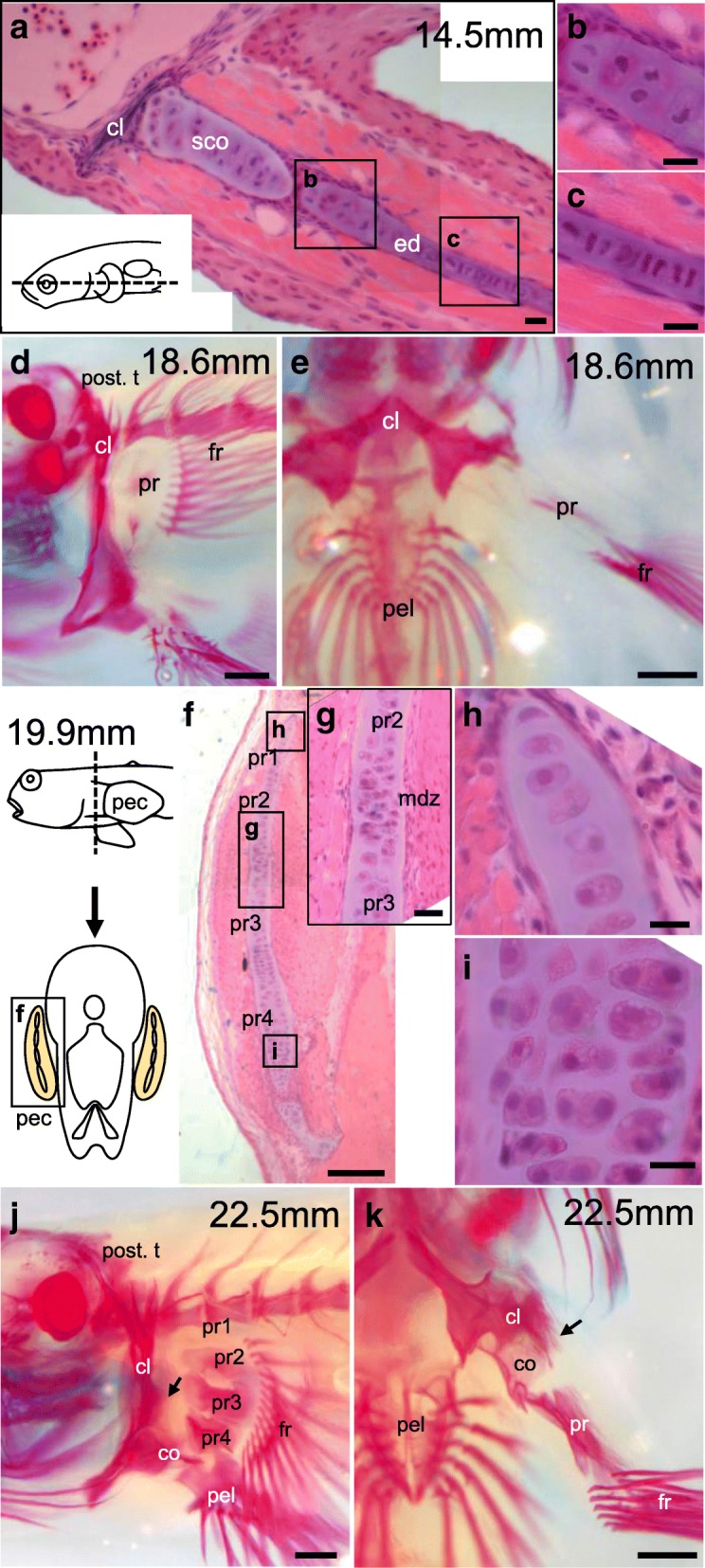
Skeletal patterns of pectoral fins of juveniles. **a**–**c** Hematoxylin- and eosin-stained pectoral fins of 14.5 mm TL planktonic larvae (*n* = 1). **b**, **c** Magnified figures indicated in (**a**). **d**, **e** Lateral (**d**) and ventral (**e**) views of skeletal patterns of pectoral fins of 18.6 mm TL benthic juveniles (*n* = 1). **f**–**i** Hematoxylin- and eosin-stained pectoral fins of 19.9 mm TL amphibious juveniles (*n* = 1). **g**, **h**, **i** Magnified figures indicated in (**f**). **j**, **k** Lateral (**j**) and ventral (**k**) views of skeletal patterns of pectoral fins of 22.5 mm TL amphibious juveniles (*n* = 1). cl, cleithrum; co, coracoid; fr, fin ray; mdz, zone of matrix decomposition; pel, pelvic bone; pt., posttemporal; pr, proximal radial; scl, supracleithrum; sco, scapulocoracoid. Scale bars, 10 μm in (**a–c**, **g**–**i**); 50 μm in (**f**); 400 μm in (**d**–**e**, **j**–**k**)

The first dorsal fin is noticeable at this stage (Additional file 3: Figure S2J). There are 14 fin rays within the fin fold of the fan-shaped pectoral fin (arrowheads in Fig. 4f). These features are similar to those found in the larvae immediately before their transition from a pelagic to benthic habitat (late post larva in Fig. 4F in Kobayashi et al., 1972). Frontal sections of the pectoral fin show that the scapulocoracoid (sco) protrudes and becomes the proximal part of the fin (Fig. 6a). The proximal region of the endoskeletal disc is two cells wide along the dorso-ventral axis (Fig. 6b), whereas it is single-cell wide in the middle region (Fig. 6c).

#### Benthic juvenile stages (Fig. 4; Fig. 6)

##### 18.6 mm TL (Fig. 4g; Fig. 6d-e; Additional file 3: Figure S2K-L)

Pelvic fins are noticeable from a lateral view, and fin rays are seen within the fin-fold region of the pelvic fins (Additional file 3: Figure S2L). Eyes begin to move dorsally (Additional file 3: Figure S2K). More melanophores are seen in the head region (arrowheads in Additional file 3: Figure S2K). The pectoral fins change from fan shape into paddle shape (Fig. 4g; Additional file 3: Figure S2L). The endoskeletal disc begins to be ossified and becomes the proximal radials (pr). Ossification is also observed in the fin rays (fr) of the pectoral fin and in the post-temporal (post. t.) (Fig. 6d). Most of the cleithrum is ossified, although the cleithrum plate (clp in Fig. 1d), a characteristic of adult fish, is not yet observed (Fig. 6d, e).

##### 19.2–19.9 mm TL (Fig. 4h-i; Fig. 6f-i; Additional file 3: Figure S2M)

Melanophores are spread over the entire body, including the pectoral fins (Fig. 4h; Additional file 3: Figure S2M). The mouth moves ventrally (Additional file 3: Figure S2M). The proximal region of the pectoral fin is covered by the well-developed operculum (ope) (Fig. 4h, i). Frontal sections of the pectoral fins show that four proximal radials (pr1–pr4) are each isolated by a zone of matrix decomposition (mdz in Fig. 6g). The first and second proximal radials are single-cell wide (Fig. 6h), whereas the third proximal radial is two cells wide and the fourth proximal radial is two- or three-cell wide along the dorso-ventral axis at the proximal part of the pectoral fin (Fig. 6i).

#### Amphibious juvenile stage (Additional file 3: Figure S2N; Fig. 6j-k)

##### 21.1–22.5 mm TL (Additional file 3: Figure S2N; Fig. 6j-k)

Eyes protrude at the top of the head, and the mouth is located on the ventral side. The rostrum becomes flattened and horizontal to the body axis (Additional file 3: Figure S2N). Melanophores cover the entire body surface. The juvenile at this stage is similar in appearance to the adult, except for the developing first dorsal fins (Additional file 3: Figure S2N) and begins to show an amphibious ecology [7].

Alizarin red– and alcian blue–stained skeletal specimens show that the ossification of the cleithrum plate (arrow in Fig. 6j, k), the first through fourth proximal radials (pr1–pr4), and the coracoid (co) has taken place (Fig. 6j, k). Skeletal patterns of the pectoral fin (Fig. 6j, k) resemble those of adult fish (Fig. 1c).

## Discussion

### Remodeling of the endoskeletal disc

**Fig. 7:**
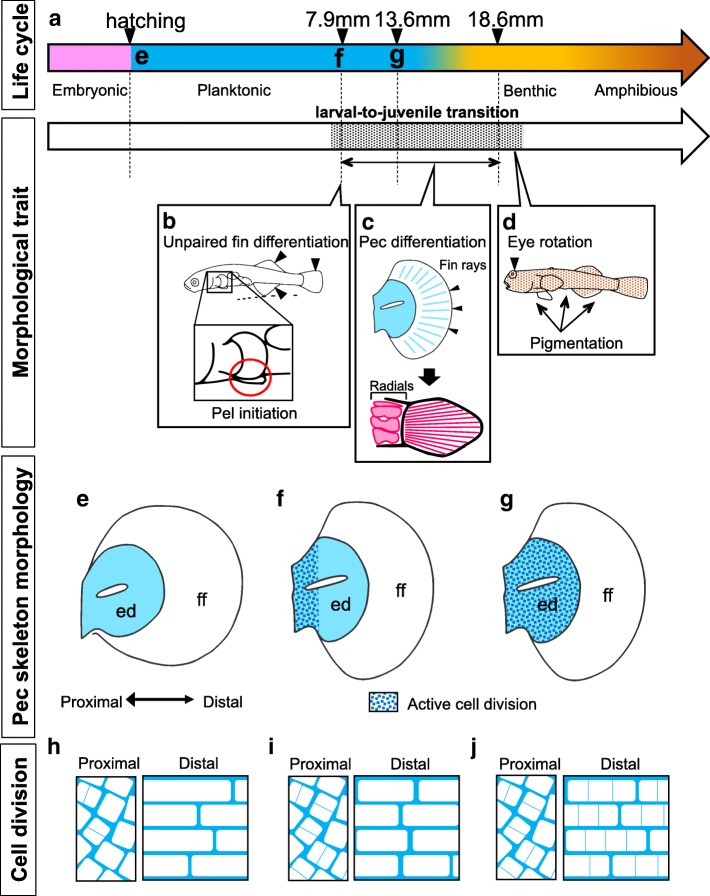
Remodeling of the pectoral girdle and endoskeletal disc during the larva-to-juvenile transition. **a** Developmental stages of the mudskipper *Ps. modestus*. Pink, blue, yellow, and brown bars indicate embryonic, planktonic larval, benthic juvenile, and amphibious juvenile stages, respectively. The shaded region indicates the larva-to-juvenile transition. **b**–**d** Morphological changes observed during the larva-to-juvenile transition. Indicative metamorphic changes are observed during 7.9 mm to 18.6 mm TL, including differentiation of the median fin and initiation of pelvic fin bud formation during 7.9 mm TL (**b**); formation of 14 fin rays during 14.5 mm TL and their ossification, as well as individualization of proximal radials and their ossification during 18.6 mm TL (**c**); and the dorsal shift in eye position and dispersion of melanophores throughout the body during 18.6 mm TL (**d**). **e**–**g** Morphological changes in the endoskeletal disc at each stage indicated in (**a**). Blue dots indicate the region where active cell divisions are observed. ed., endoskeletal disc: ff, fin fold. **h**-**j** The shape and oriented division of chondrogenic cells of the endoskeletal disc within the pectoral fin. **h** At the beginning of the planktonic larval stage, round-shaped cells are found proximally, whereas cylindrical cells are aligned along the proximal-distal axis. **i** At the beginning of the larva-to-juvenile transition [10], cells are actively dividing in the proximal region, whereas in the distal region they are cylindrical in shape, only infrequently dividing, and aligned along the proximal-distal axis. **j** At the end of the larva-to-juvenile transition, the distal cylindrical cells are dividing actively along the proximal-distal axis. See text for details

In this study, we found that the shape of the endoskeletal disc transforms from elliptical (Fig. 7e) to fan-shaped (Fig. 7f) during metamorphosis from larvae (7.9 mm TL) to juveniles (13.6 mm TL) in the mudskipper *Ps. modestus*, which corresponds to the larva-to-juvenile transition (metamorphosis).

In teleost fishes, various morphological changes are induced during metamorphosis, including initiation of pelvic fin bud formation, differentiation of the pectoral fin bud, changes in the pattern of pigmentation, a shift in eye position, and differentiation of the median fin [12–14]. We observed the differentiation of the median fin to the dorsal, caudal, and anal fin and an initiation of the pelvic fin bud at 7.9 mm TL (Fig. 7b). Fourteen fin rays are formed in the pectoral fin by 14.5 mm TL, and subsequently they are ossified by 18.6 mm TL (Fig. 7c). A dorsal shift in the eyes, ossification of the proximal radials, and differentiation of melanophores are also observed during this stage (Fig. 7c, d). Therefore, it seems that the larva-to-juvenile transition occurs during 7.9 mm to 18.6 mm TL, when mudskippers shift from planktonic to benthic lifestyle.

During the larva-to-juvenile transition, the shape of the endoskeletal disc likely is remodeled by the localized cell division occurring in the proximal region (Fig. 7e, f, h, i) and the subsequent oriented cell divisions along the proximal-distal axis that are restricted to the distal region (Fig. 7g, j). Such localized and oriented cell divisions are not observed in zebrafish [11]. Thus, localized and oriented cell divisions in the endoskeletal disc seem to create the elongated proximal radials, one of the defining characteristics of the mudskipper pectoral fin. Kinematic analyses of mudskippers show that such elongated radials enable the pectoral fins to move forward for a longer distance per a single stroke cycle [1, 4] and also provide more powerful driving force by making a larger surface onto which pectoral muscles can connect [1, 2, 4].

We found that a major characteristic of the pectoral skeletal pattern of mudskippers, i.e., the elongation of the proximal radials, is formed during the larva-to-juvenile transition. Various morphological changes that occur during this metamorphic period are dependent on thyroid hormones in many teleost fishes [12, 13]. Zebrafish larvae treated with thyroid hormones exhibit the mature pectoral fin morphology and elongation of the pelvic fin bud [12]. In contrast, inhibition of thyroid hormone synthesis results in the suppression of the maturation of pectoral and pelvic fins [12]. However, treatment with thyroid hormone alone cannot complete the larva-to-juvenile transition [12], suggesting that other factor(s) are also involved in this metamorphosis step. Future studies should clarify what factors are involved in the remodeling of pectoral skeletal elements during metamorphosis.

### The preaxial proximal radial

The preaxial proximal radial of the mudskipper pectoral fin is longer than the postaxial radial [2]. Harris suggested that such a difference in the length of proximal radials consequently lowers the fin axis below the horizontal and thus places the fin rays of mudskippers in a more effective position for transmitting their force to the ground [2].

Here we showed that two main morphological characteristics of the pectoral fins that are unique to mudskippers are formed during the larva-to-juvenile transition, whereas the developmental processes of pectoral fin buds of mudskippers during embryogenesis and larval development do not seem to be modified as compared with those of zebrafish [11, 15]. During zebrafish embryogenesis, pectoral fin buds grow along their proximal-distal axis, which is parallel to the dorso-ventral body axis [11, 15]. As pectoral fin buds grow, they rotate from the initial position in a way that allows their distal margins to point caudally and their anterior side to face upwards with respect to the body axis [11, 15]. Immediately before this rotation, chondrogenic condensation begins to form the endoskeletal disc, and the apical ectodermal thickening becomes transformed into the apical fin fold [11, 15]. When the rotation of the pectoral fin bud is complete, a single-cell-layered endoskeletal disc and the apical fin fold structure become apparent [11, 15]. Similarly, during the embryogenesis of the mudskipper, pectoral fin buds begin to rotate their initial position and point their distal tips caudally (Fig. 2e, f). At the same time, the apical fin folds are seen at the distal tips of pectoral fin buds (Fig. 2e, f). Chondrogenic condensation also begins within the pectoral fin buds just before the rotation of the fin (Fig. 3b) and becomes a single-cell-layered endoskeletal disc after this rotation (Fig. 3c). These results suggest that developmental processes of the mudskipper pectoral fin bud during embryogenesis and larval development are similar to those of zebrafish [11, 15], and thus the unique characteristics of mudskipper pectoral fins do not form before the larva-to-juvenile transition.

## Conclusions

Here we found that the localized cell division of the proximal part of the endoskeletal disc and subsequent cell divisions along the proximal-distal axis specifically in the distal region during metamorphosis lead to the protrusion and elongation of the proximal radials. Future studies are needed to further explore whether these metamorphic changes are observed during development of the pectoral fins in various mudskippers as well as in the rest of the oxudercine gobies to understand how mudskippers evolved their pectoral fin morphology to adapt to a terrestrial environment.

## Additional files

Additional file 1: **Figure S1.** Development of mudskipper embryos before hatching. (A–V) Mudskipper embryos at the otic vesicle stage (A–C; *n* = 2), brain vesicle stage (D–F; *n* = 2), heart chamber stage (G–I); *n* = 2, circulation stage (J, K; n = 2), eye pigmentation stage (L, M; n = 2), middle-pec stage (N, O; *n* = 2), high-pec stage (P, Q; *n* = 2), long fin-fold stage (R-T; *n* = 2), and hatching stage (U, V; *n* = 2). Lateral views of the embryo (A, D, G, J, L, N, P, R, U), the head (B, E, S, V), the tail (C, F, I, T), the trunk (H, O, Q), the eye (M), and the posterior region of the yolk (K, O). (E’, S′) Magnified images of the otic vesicle indicated in (E) and (S), respectively. (G’) A magnified image of the tail region of the same embryo as in (G). Arrowheads in (F, M) indicate melanophores and in (K) indicate blood cells. Open arrowheads in (E, F, G, M) indicate xanthophores. at, atrium; ccv, common cardinal vein; d, diencephalon; h, heart; lj, lower jaw; lp, lens primordium; mc, mesenchymal cell; mf, median fin; m, midbrain; nc, notochord; op, optic vesicle; ot, otolith; ov, otic vesicle; r, rhombomere; sb, swim bladder; t, telencephalon; uj, upper jaw; vt, ventricular. Scale bars, 100 μm in A-V, G’; 10 μm in E’, S′. (PDF 484 kb)

Additional file 2: **Table S1.** Comparison of the stages of *Periophthalmus modestus* in this study with those suggested by Kobayashi et al. (1972). (PDF 164 kb)

Additional file 3: **Figure S2.** Development of mudskipper larvae and juveniles after hatching. (A–N) Lateral views (A–D, E, G, I, J–N) and ventral views (F, H) of planktonic larvae at 4.0–11.0 mm TL (A–H; *n* = 1 each stage) and of benthic to amphibious juveniles at 14.5–21.1 mm TL (I-N; *n* = 1 each stage). (J, L) Magnified images. Arrowheads in (D) indicate fin rays in the caudal fin. Arrows in (D, E) and arrowheads in (G, I, K) indicate the primordia of the hypural and melanophores, respectively. af, anal fin; cf., caudal fin; df1, dorsal fin 1; df2, dorsal fin 2; lj, lower jaw; mf, medial fin; pec, pectoral fin; pel, pelvic fin; y, yolk. Scale bars, 100 μm in D, F, H; 500 μm in A-C, J; 1 mm in E, G, I, K–N. (PDF 311 kb)

## References

[1] Pace CM, Gibb AC (2014). Sustained periodic terrestrial locomotion in air-breathing fishes. J Fish Biol.

[2] Harris VA (1960). On the locomotion of the mud-skipper Periophthalmus koelreuteri (Pallas): (Gobiidae). Proc Zool Soc Lond.

[3] Murdy EO. A taxonomic revision and Cladistic analysis of the Oxudercine gobies (Gobiidae: Oxudercinae). Rec Aus Mus 1989. Suppl 11:1–93.

[4] Pace CM, Gibb AC (2009). Mudskipper pectoral fin kinematics in aquatic and terrestrial environments. J Exp Biol.

[5] Eggert B (1929). Bestimmungstabelle und Beschreibung des Arten der family Periophthalmus. Z Wiss Zool.

[6] Ishimatsu A, Yoshida Y, Itoki N, Takeda T, Lee HJ, Graham JB (2007). Mudskippers brood their eggs in air but submerge them for hatching. J Exp Biol.

[7] Kobayashi T, Dotsu Y, Miura N (1972). Egg development and rearing experiments of the larvae of the muds skipper, Periophthalmus cantonesis. Bull Fac Fish, Nagasaki Univ.

[8] Walker MB, Kimmel CB (2007). A two-color acid-free cartilage and bone stain for zebrafish larvae. Biotech Histochem.

[9] Kaneko H, Nakatani Y, Fujimura K, Tanaka M (2014). Development of the lateral plate mesoderm in medaka Oryzias latipes and Nile tilapia Oreochromis niloticus: insight into the diversification of pelvic fin position. J Anat.

[10] Liu YW, Chan WK (2002). Thyroid hormones are important for embryonic to larval transitory phase in zebrafish. Diff.

[11] Grandel H, Schulte-Merker S (1998). The development of the paired fins in the zebrafish (Danio rerio). Mech Dev.

[12] Brown DD (1997). The role of thyroid hormone in zebrafish and axolotl development. Proc Natl Acad Sci U S A.

[13] McMenamin SK, Bain EJ, McCann AE, Patterson LB, Eom DS, Waller ZP, Hamill JC, Kuhlman JA, Eisen JS, Parichy DM (2014). Thyroid hormone-dependent adult pigment cell lineage and pattern in zebrafish. Science.

[14] Miwa S, Inui Y (1987). Effects of various doses of thyroxine and triiodothyronine on the metamorphosis of flounder (Paralichthys olivaceus). Gen Comp Endocrinol.

[15] Kimmel CB, Ballard WW, Kimmel SR, Ullmann B, Schilling TF (1995). Stages of embryonic development of the zebrafish. Dev Dyn.

