# Supplementary figures and images for: Modification of pectoral fins occurs during the larva-to-juvenile transition in the mudskipper (Periophthalmus modestus)
Source: Zoological Lett. 2018 Aug 11;4:23. doi: 10.1186/s40851-018-0105-z (PMC6086994; doi:10.1186/s40851-018-0105-z)

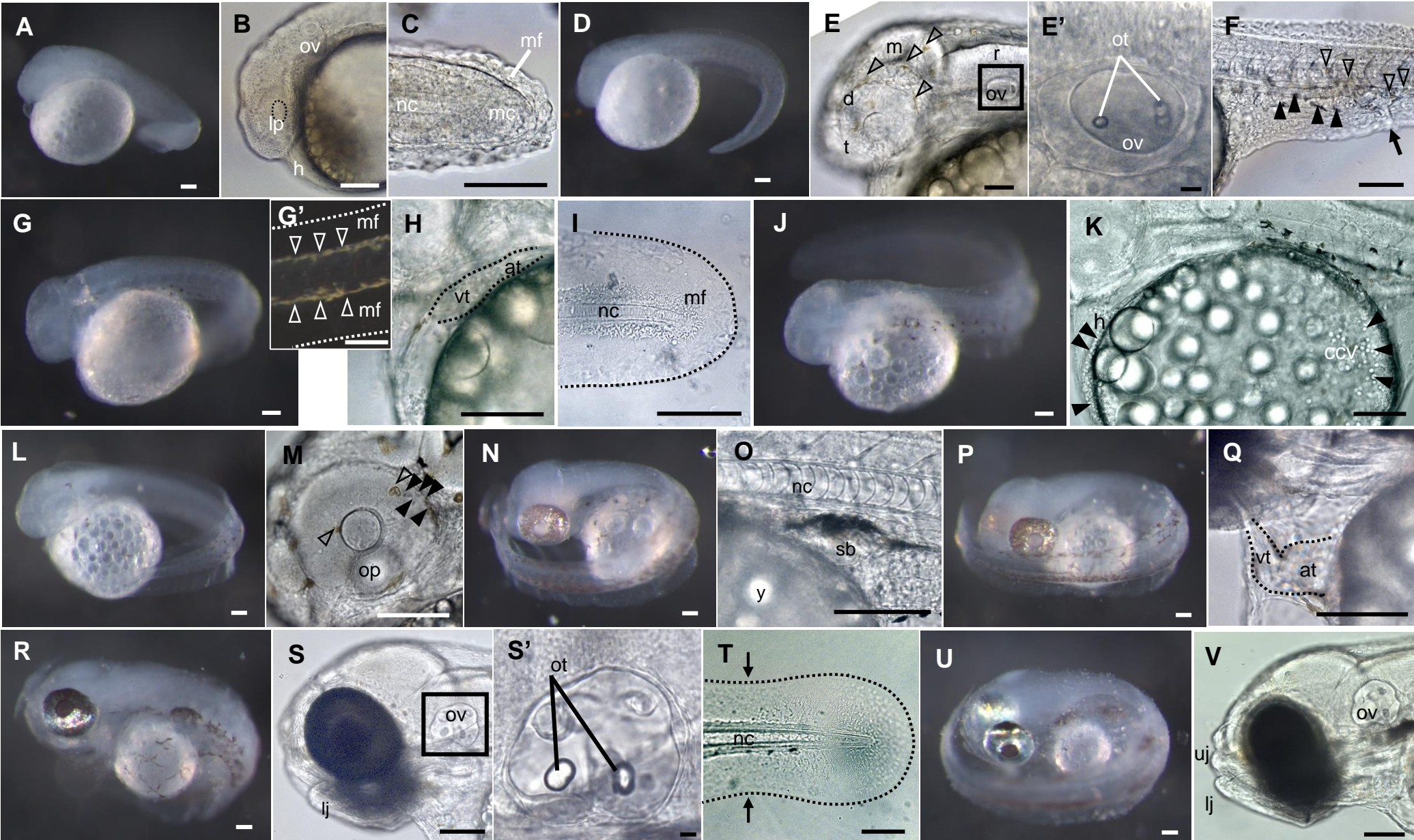

Okamoto et al. Supplemental Fig. 1

Supplement: Supplementary file 1 — Figure S1. Development of mudskipper embryos before hatching. (A–V) Mudskipper embryos at the otic vesicle stage (A–C; n = 2), brain vesicle stage (D–F; n = 2), heart chamber stage (G–I); n = 2, circulation stage (J, K; n = 2), eye pigmentation stage (L, M; n = 2), middle-pec stage (N, O; n = 2), high-pec stage (P, Q; n = 2), long fin-fold stage (R-T; n = 2), and hatching stage (U, V; n = 2). Lateral views of the embryo (A, D, G, J, L, N, P, R, U), the head (B, E, S, V), the tail (C, F, I, T), the trunk (H, O, Q), the eye (M), and the posterior region of the yolk (K, O). (E’, S′) Magnified images of the otic vesicle indicated in (E) and (S), respectively. (G’) A magnified image of the tail region of the same embryo as in (G). Arrowheads in (F, M) indicate melanophores and in (K) indicate blood cells. Open arrowheads in (E, F, G, M) indicate xanthophores. at, atrium; ccv, common cardinal vein; d, diencephalon; h, heart; lj, lower jaw; lp, lens primordium; mc, mesenchymal cell; mf, median fin; m, midbrain; nc, notochord; op, optic vesicle; ot, otolith; ov, otic vesicle; r, rhombomere; sb, swim bladder; t, telencephalon; uj, upper jaw; vt, ventricular. Scale bars, 100 μm in A-V, G’; 10 μm in E’, S′. (PDF 484 kb) [file 40851_2018_105_MOESM1_ESM.pdf]

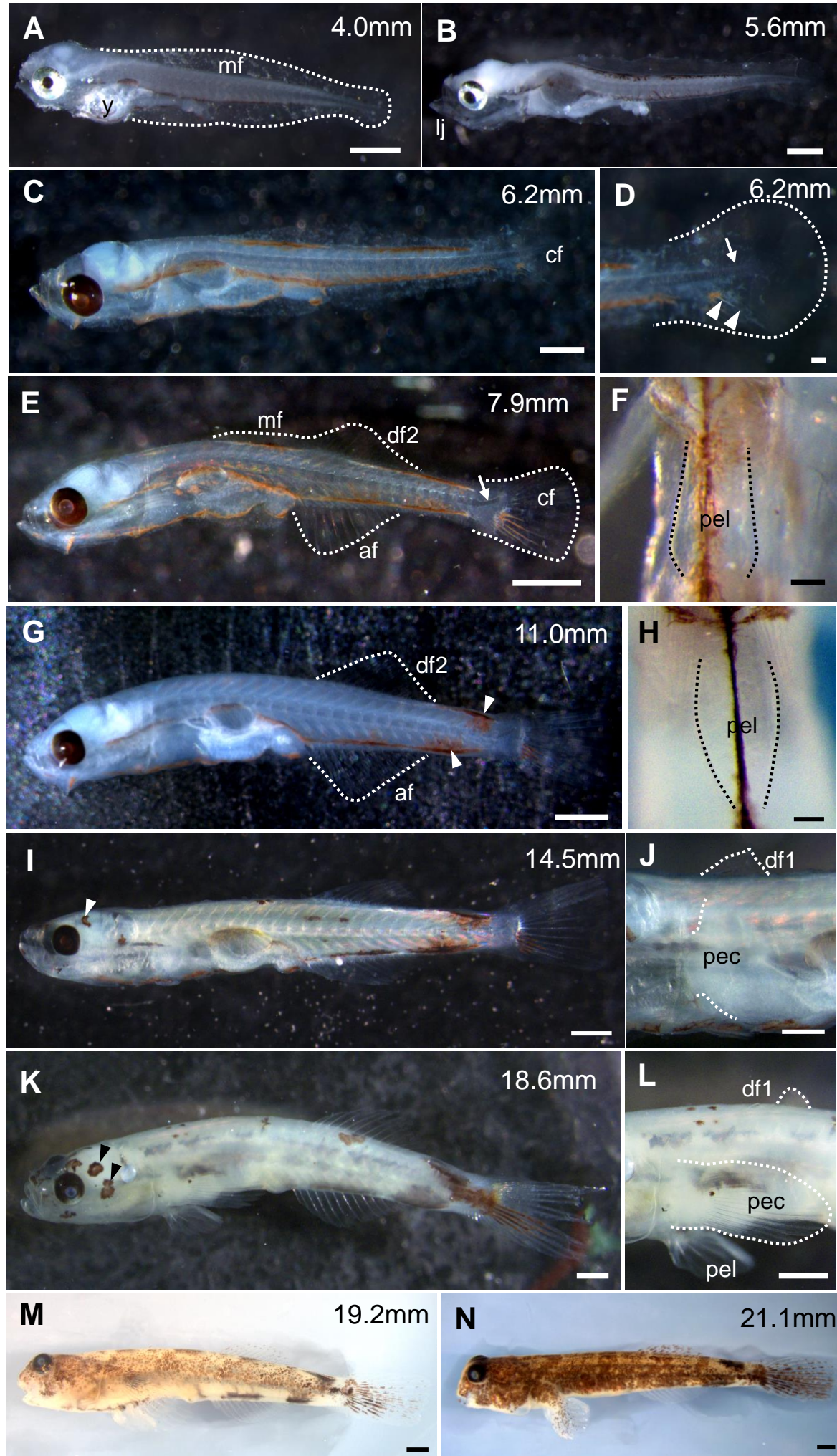

Okamoto et al. Supplemental Fig. 2

Supplement: Supplementary file 3 — Figure S2. Development of mudskipper larvae and juveniles after hatching. (A–N) Lateral views (A–D, E, G, I, J–N) and ventral views (F, H) of planktonic larvae at 4.0–11.0 mm TL (A–H; n = 1 each stage) and of benthic to amphibious juveniles at 14.5–21.1 mm TL (I-N; n = 1 each stage). (J, L) Magnified images. Arrowheads in (D) indicate fin rays in the caudal fin. Arrows in (D, E) and arrowheads in (G, I, K) indicate the primordia of the hypural and melanophores, respectively. af, anal fin; cf., caudal fin; df1, dorsal fin 1; df2, dorsal fin 2; lj, lower jaw; mf, medial fin; pec, pectoral fin; pel, pelvic fin; y, yolk. Scale bars, 100 μm in D, F, H; 500 μm in A-C, J; 1 mm in E, G, I, K–N. (PDF 311 kb) [file 40851_2018_105_MOESM3_ESM.pdf]
