# Supplementary material for: Modification of pectoral fins occurs during the larva-to-juvenile transition in the mudskipper (Periophthalmus modestus)
Source: Zoological Lett. 2018 Aug 11;4:23. doi: 10.1186/s40851-018-0105-z (PMC6086994; doi:10.1186/s40851-018-0105-z)
Supplement: Supplementary file 2 — Table S1. Comparison of the stages of Periophthalmus modestus in this study with those suggested by Kobayashi et al. (1972). (PDF 164 kb) [file 40851_2018_105_MOESM2_ESM.pdf]

Supplemental Table 1. Comparison of the stages of *Periophthalmus modestus* in this study with those suggested by Kobayashi et al. (1972).

| | **Kobayashi et al., 1972** | | **This study** | |
|---|---|---|---|---|
| Rearing condition | ・Artificial insemination<br>・Incubated at 19-20℃ in 50% sea water | | ・Collected naturally fertilized egg<br>・Incubated at 24-26℃ in air | |
| Stage correspondence table | Embryonic stage classification | hours after fertilization | Embryonic stage classification | hours after otic vesicle stage |
| | Fig. 1L | 39-46h | Otic vesicle stage | 0h |
| | Fig. 1M-N | 52-63h | Brain vesicle stage | 6h |
| | | | Heart chamber stage | 8.5h |
| | | | Circulation stage | 11.5h |
| | Fig. 1O | 99h | Eye pigment stage | 20h |
| | | | Low-pec stage | 26h |
| | Fig. 1P | 117-118h | Middle-pec stage | 34.5h |
| | | | High-pec stage | 39.5h |
| | | | Long pec stage | 44.5h |
| | | | Middle-fin fold stage | 55.5h |
| | | | Long-fin fold stage | 60h |
| | Fig.1Q | 170-175h | Hatching stage | 78h |
